# Subcostal wire migration from the left clavicle internal fixation: A case report

**DOI:** 10.1016/j.ijscr.2024.109998

**Published:** 2024-07-05

**Authors:** Meirizal, Muhammad Fadhil Wasi Pradipta, Agung Susilo Lo, A Faiz Huwaidi

**Affiliations:** a Department of Orthopedics and Traumatology, RSUP Dr. Sardjito Hospital, Jl. Kesehatan Sendowo No.1, Sleman 55281, D.I.Yogyakarta, Indonesia; b Faculty of Medicine, Public Health and Nursing, Universitas Gadjah Mada, Jl. Farmako, Sendowo, Sekip Utara, Sleman 55281, D.I.Yogyakarta, Indonesia

**Keywords:** Kirschner wire, Migration of K-wire, Clavicle fracture

## Abstract

**Background:**

Kirschner wire is a widely used implant in orthopedics, with migration being a typical problem following internal fixation. Subcostal wire migration might result in catastrophic problems such as penetration of the heart, lungs, trachea, big blood vessels, or abdominal cavity. Every orthopedic surgeon must be vigilant and mindful of the potential hazards of wire migration.

**Case report:**

a 45-year-old Indonesian male was referred from another hospital. 1 year prior, the patient underwent internal fixation of the left clavicle. 2 weeks before admission, the patient complained of stabbing pain in the left shoulder area. An X-ray examination revealed a broken end wire in the left infraclavicular area. Immediate wire-extraction surgery was planned. An X-ray and CT scan showed that the wire had migrated into the left lateral side of the 9th subcostal space and was heading inferiorly. The Thoracic and Cardiovascular Surgery Department carried out the wire evacuation. The wire was successfully removed without any concern.

**Discussion:**

Previous studies have suggested that wire migration can occur due to muscular activity, respiratory motion, gravity, and upper-extremity movement. Wire migration is a condition that can occur following shoulder fixation, especially in comminuted fractures that typically use K-wires to stabilize the fragments. Upon the detection of wire migration, prompt evacuation should be conducted to mitigate the severity.

**Conclusion:**

In cases of wire migration, orthopedic surgeons should pay special attention. Actions that can be taken to prevent wire migration are to: bend the wire, use a threaded wire, and remove it quickly after callus formation.

## Highlights

• Wire migration is a frequent complication following the internal fixation of clavicle fractures.
• Motion around fracture site can cause K-wire migration, bending with an angle of 180 degree would be an anchor for K-wire to prevent migration
• A threaded wire, such as a Schanz screw, can be used to improve bone traction and reduce the likelihood of migration.
• The K-wire should be removed immediately if fracture or migration is detected, regardless of the absence of symptoms.

## 1 Introduction

Clavicle fractures are frequently observed in clinical settings and account for between 2.6 and 10 % of all fractures in the body. More than 80 % of clavicle fractures often occur in the central section of the clavicle, while fractures in the distal and proximal sections are less frequent [23,24]. Mid-shaft clavicle fractures can present in various forms, ranging from simple transverse fractures to more complex comminuted fractures. The primary objective in managing clavicle fractures is to immediately restore capability to the upper limb and prevent any disability. Patients with completely displaced mid-third clavicle fractures often experienced favorable outcomes. However, there was a 15 % difference that was strongly associated with an unsatisfactory outcome. Recent studies suggest that the likelihood of nonunion is significantly higher when a clavicle fracture causes more than 2 cm of bone shortening or over 100 % displacement [1]. Surgical intervention enhances the prognosis of these fractures [16]. Performing surgery to minimize soft tissue damage and stabilizing bone fragments presents a significant difficulty in preventing non-union [2,6].

Surgical procedures for middle third clavicle fracture range from plate and screw, intramedullary wire, and cerclage wire. The wire is a potential option for clavicle fixation among many implants. Cerclage wire is commonly employed to stabilize fragmented bone pieces in the clavicle. A study by Nakayama et al. on clavicle fracture surgery included a combination of implants and wires for reducing damage to soft tissues [10]. The study found that temporary axial K-wire fixation can effectively resolve the difficulties associated with unstable clavicle fractures and produce a successful fracture reduction [2,10]. However, in the current era, the use of pinning on the clavicle has been largely abandoned due to the high risk of displacement, and malunion migration in clavicle fixation. On the other hand, plate and screw fixation is often preferred over wire fixation due to its potential efficacy in achieving optimal outcomes [22].

When selecting wire as an implant choice, it is important to carefully consider the potential issues associated with its use. Wire migration is a frequent complication following the internal fixation of clavicle fractures [8,9]. The phenomenon of wire migration following shoulder procedures and the subsequent consequences are widely recognized. In our cases, the migration happens in a forward direction and may cause harm. The thoracic cavity, containing the essential organs, poses the greatest risk of life-threatening wire migration. We deliberated on the diagnosis, surgical intervention, and post-operative result. Given the lack of research on the migration of subcostal K-wires following surgery, this specific study would provide significant advantages to both academics and physicians. The writing of this case report complied with the SCARE criteria [36].

## 2 Case presentation

**Fig. 1:**
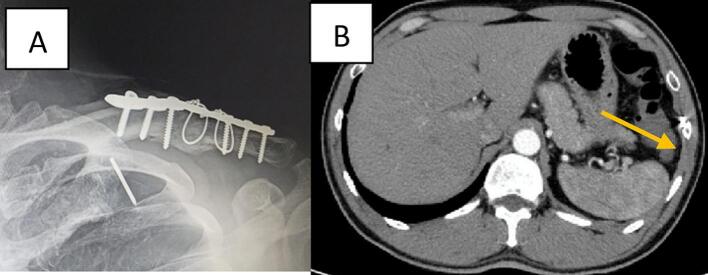
(A) Post-operative X-ray. (B) CT – Scan evaluation show the wire position below 9th costae with no organ damage.

An Indonesian male patient 45 years old, was referred from another hospital to the outpatient clinic with acute shoulder pain characterized by a stabbing sensation. The condition manifested about two weeks prior to the consultation. The patient's medical record indicated that he had previously open reduction and internal fixation with K wire for a left clavicle fracture about a year ago. The patient's vital signs were stable on physical examination and pain when palpation in the left infraclavicular region. An X-ray of the chest revealed an opaque, wire-shaped foreign body with 2 × 1 cm in size, located vertically in the 1st intercostal space. There are no signs of associated complications such as pneumothorax or vascular injury (Fig. 1A).

Removal of the wire was recommended, and the patient was hospitalized in preparation for surgery. However, during thorax evaluation, the wire migrated to the left subcostal area (Fig. 1B). Chest computerized tomography revealed the metallic foreign body was in the left 9th subcostal region outside of the mediastinum. There was no evidence of organ damage. Immediate surgery was planned together with the cardiothoracic and vascular surgeons.

**Fig. 2:**
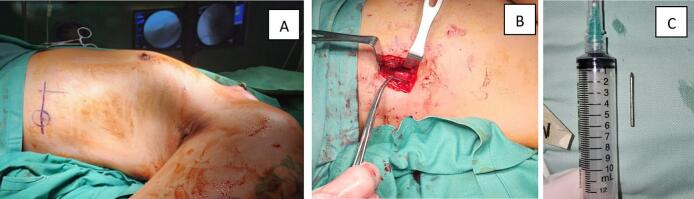
(A) Mid-axilla incision for removal wire. (B) Wire located at the subcostal region with no penetration organ damage. (C) Unbend straight wire size 2.5 cm.

A 10 cm vertical incision at the level of the 9th subcostal region was performed and deepened until a wire was found located between the diaphragm and the 9th costae. Exploration of adjacent structures found no sign of organ damage or penetration through the thoracic wall. Since the wire was linear in shape, it was easy to remove. The unbent entire K-wire, which was 2.5 cm with a diameter of 1.6 mm, is removed **(**Fig. 2**)**. We ensure there is no injury to the pleural cavity by carrying out a water seal test (submersion test). By pouring an isotonic NaCl into the operating area, we do not observe bubble water. Then the remaining implant and wire on the left clavicle were removed. The total intraoperative blood loss was approximately 100 ml. On the third postoperative day, a follow-up examination of the chest was performed. The patient recovered without complications and was discharged on the third postoperative day. At the 3-month follow-up, the patient had no specific symptoms such as cough or chest pain. The clavicle fracture had union without any complications.

## 3 Discussion

Compared to other fractures, clavicle fractures are more common than other fractures, with an incidence of 2.6 %–10 % with the middle third as the most affected part with a prevalence of 80 of the cases. The central portion of the clavicle is the narrowest and most susceptible to fractures among all sections of the collarbone. Both surgical and non-operative treatment methods are available for clavicle fractures [25]. The primary objective of clavicular fracture treatment is to promptly recover the functionality of the upper limb and prevent any form of impairment [26]. Historically, the vast majority of fractures occurring in the middle section of the clavicle may be managed without the need for surgery. The non-surgical approach involves immobilization using a sling or figure-of-eight bracing treatment [27]. Nevertheless, adopting a conservative approach may have been associated with unfavorable clinical outcomes and increased comorbidities.

Early surgical intervention is essential for the successful treatment of mid-clavicle fractures, especially those that are indicated. Surgical treatment options for clavicle fractures include internal fixation with the insertion of intramedullary metal wire or a metal plate. When choosing a suitable method of fixation, it is important to consider various factors related to the patient, including their age, level of physical activity, hand dominance, the pattern of the fracture, and the surgeon's preference for utilizing a particular implant. A frequently employed implant for stabilizing clavicle fractures, the K-wire was known for its efficacy in securing small fragments of bone, perceived minimal soft tissue injury profile, and ability to maintain blood supply to the bone [[5], [6], [7]]. Despite being acknowledged for its potential advantages, the utilization of k-wire for clavicle fixation remains a subject of controversy. A comparative study examining the utilization of wire and plate screws revealed no substantial link between the duration of operation and blood loss [22]. There were even significant side effects from using wires, ranging from the risk of fragment displacement, and malunion to a high risk of wire migration [28,29].

Wire migration is a typical problem that occurs after using K-wires to treat clavicle fractures or shoulder dislocations. We cited four instances of intracardiac pin migration as a surgical complication in the shoulder region. Later, several cases were documented in the literature. K-wire fixation of the sternoclavicular joint was the primary site of migration to the heart, while the clavicle, acromioclavicular joint, and proximal humerus were secondary sites [3,8,10,13,14]. K-wire migration from the lower extremities to the heart has been reported. The precise process of K-wire migration remains uncertain, however, in our case, we believe that it to occur at a very gradual pace. Several theories have been suggested to clarify the cause of this migration, such as muscular activity, joint mobility, respiratory movements, negative pressure within the chest, and localized bone reabsorption without bending or threading of the pin [8,9,11,12]. Possible causes of K-wire subcostal migration in our instance may be attributed to movements of the gravity, and thoracoclavicular joint, subsequently influenced by respiratory movements. According to our research of literature, we conclude that the patient's occupation as a construction worker resulted in arm movements that put a heavy strain on the clavicle fixation. This strain led the wire to break and slide toward the shoulder region [30,31].

Based on all these considerations, we strongly believe that the root cause of K-wire displacement is K-wire bending. This mechanism is a reminder that K-wire should be used with caution in amphiarthrosis unless warranted. Regardless of the method used, the K-wire can reach every possible place in the body and cause various difficulties. The chest is the most dangerous place for wire migration. If any indication of migration is observed, it is advisable to consider surgical intervention, as the presence of the metallic wire may lead to lung penetration, heart perforation, and potentially fatal consequences [14]. Straight wire is highly susceptible to rapid displacement due to the absence of resistance that would enable it to adhere. In this case, we observed wire migration in the infraclavicular region, which then migrated to the subcostal area within 3 days. This highlights the significance of promptly monitoring and treating cases of wire migration.

To prevent K-wire movement, the surgeon must follow the procedure for handling K-wire implants [12,32,34]. Movement at the fracture site can cause the K-wire to shift. A 180-degree bend in the K-wire creates an anchor that ensures smooth insertion into the bone and provides stable fixation [21]. Threaded wire, such as the Schanz Screw, can be utilized to enhance the grip in bone and minimize the likelihood of migration [32,33]. Nevertheless, the use of threading poses risks to migration situations if it is employed for an extended period of time [34]. Another factor to take into account is that K-wires should be extracted once they have successfully achieved their intended purpose of temporary fixation during the surgical procedure. Nevertheless, there remains a potential for the K-wires to become loose or break during the postoperative period as a result of osteopenia or joint movement [13,14]. A complete X-ray examination and regular clinical follow-up every 2–4 weeks are necessary to assess the progress of bone healing and the position of internal fixation. The K-wire should be promptly removed if it is discovered to be damaged, or moving, or once bone healing is accomplished during the 4–6 month follow-up period [13,14].

In addition to the aforementioned preventive measures, it may be advisable to utilize plates and screws for clavicle fixation [19,35]. In cases where there are little pieces that do not compromise the bone's structural integrity, they can be retained. This procedure is superior because it can yield equivalent functional results, such as fracture healing and joint function recovery, but with a reduced duration of surgery [19,35].

## 4 Conclusion

The research highlights the hazards associated with the use of K-wire for the fixation of clavicle fractures. Surgeons should be aware of the possibility of significant complications resulting from K-wire migration. To minimize the possibility of migration, it is recommended to (1) utilize K-wires with threaded tips, and (2) bend-free ends. (2) Timely removal of fixation is necessary once the fracture is deemed stable [15]. (4) The K-wire should be promptly removed upon identification of migration or fracture, regardless of the absence of any symptoms [16]. Plates and screws are a preferable way of fixation compared to wire due to their minimal dangers and equivalent effectiveness [35].

## Consent for publications

Written informed consent was obtained from the patient for publication of this case report and any accompanying images. A copy of the written consent is available for review by the Editor-in-Chief of this journal.

## Ethical approval

This case report doesn't require ethical approval based on the Universitas Gadjah Mada research ethics committee's guidelines. It focuses on a patient's treatment and medical care, not research. Our institution's ethics committee confirmed that this report aligns with routine clinical practice and doesn't involve experimental interventions or additional data collection. We're ready to provide more information if needed, underscoring our commitment to ethical practices.

## Funding

This report received no specific grant from any funding agency in the public, commercial, or not-for- profit sectors.

## Author contribution

1. M: Conceptualization, Methodology, Writing - Original Draft, Investigation, Supervision, Validation
2. M.F.W.P: Conceptualization, Methodology, Writing - Original Draft, Investigation
3. A.S. L: Conceptualization, Methodology, Writing - Original Draft
4. A.F.H: Conceptualization, Methodology, Writing - Original Draft

## Guarantor

Merizal.

## Conflict of interest statement

The authors declare no conflict of interest.

## Data Availability

Supporting data will be available upon reasonable request.
